# Methotrexate-induced leukoencephalopathy presenting as acute-onset limb weakness in a child: a case report

**DOI:** 10.1186/s13256-024-04824-5

**Published:** 2024-09-28

**Authors:** Hashan Pathiraja, Gayathri de Abrew, Linushika de Silva, Sanjaya Fernando, Shobhavi Randeny, Sachith Mettananda

**Affiliations:** 1 Colombo North Teaching Hospital, Ragama, Sri Lanka; 2 Department of Paediatrics, Faculty of Medicine, University of Kelaniya, Thalagolla Road, Ragama, 11010 Sri Lanka

**Keywords:** Methotrexate, Leukoencephalopathy, Acute limb weakness, Acute leukemia

## Abstract

**Background:**

Methotrexate is an essential medicine used to treat childhood malignancies including acute lymphoblastic leukemia. Neurotoxicity manifesting as leukoencephalopathy is an important adverse effect of methotrexate. Methotrexate-induced leukoencephalopathy classically demonstrates sub-acute-onset neurological manifestations that include learning disability, progressive dementia, drowsiness, seizures, ataxia, and hemiparesis. These are rare in children and are generally reported following intrathecal or intravenous use of methotrexate. In contrast, acute onset neurotoxicity with oral use of methotrexate is very rare. We report a 10-year-old boy presenting with acute onset limb weakness and neurological signs due to methotrexate-induced leukoencephalopathy following oral methotrexate.

**Case presentation:**

A 10-year-old Sri Lankan boy presented with fever and headache for 5 days and difficulty in walking for 2 days. He was unable to stand unaided on admission, and his parents complained of repetitive, involuntary extension movements involving the right upper limb. He is a child diagnosed with acute lymphoblastic leukemia who was on treatment for a relapse with daily oral dexamethasone and mercaptopurine, weekly oral methotrexate and folinic acid, and once every two weeks intrathecal vincristine.

On examination, he had dystonic movements of the right upper limb and hypotonia and reduced muscle power (grade 3/5) of the left upper and lower limbs proximally and distally. The muscle power of the right side was grade 4 (out of 5). Tendon reflexes were diminished in all four limbs, and the plantar response was flexor bilaterally. The child had dysmetria and intension tremors on both sides.

T2-weighted magnetic resonance imaging of the brain revealed symmetrical high signal intensities with diffusion restriction involving bilateral putamen, subcortical areas, and deep white matter, suggesting treatment-related neurotoxicity due to methotrexate-induced leukoencephalopathy. Oral methotrexate was discontinued. He showed gradual improvement in limb weakness and other neurological signs following treatment with intravenous folinic acid, aminophylline, dexamethasone, and oral dextromethorphan.

**Conclusion:**

This case report describes a patient with rapidly progressing methotrexate-induced leukoencephalopathy following oral methotrexate. It highlights that the risk of neurotoxicity persists even with the oral use of methotrexate; therefore, the prescribers should be vigilant of this uncommon side effect.

## Background

Methotrexate is an essential medicine used to treat childhood malignancies [1]. It is indicated for many cancers, including acute lymphoblastic leukemia, non-Hodgkin lymphoma, and osteosarcoma. One important adverse effect of methotrexate is neurotoxicity [2]. Neurotoxicity related to methotrexate manifests as aseptic meningitis, transverse myelopathy, encephalopathy, and leukoencephalopathy [2].

The pathophysiology of methotrexate-induced leukoencephalopathy is multifactorial. The neurotoxic effects of methotrexate are primarily attributed to the anti-folate properties of the drug, which lead to reduced synthesis of tetrahydrofolate by inhibiting dihydrofolate reductase. This leads to defects in myelination through activation of microglia and depletion of oligodendrocyte precursor cells [3]. In addition, genetic background, vitamin B_12_ deficiency, cranial irradiation, and patient predilections are reported to increase the incidence of leukoencephalopathy [4].

Methotrexate-induced leukoencephalopathy classically demonstrates sub-acute-onset neurological deficits [5]. Its manifestations include learning disability, progressive dementia, drowsiness, seizures, ataxia, and hemiparesis. These are rare in children and are generally reported following intrathecal or intravenous use of methotrexate [6]. In contrast, acute onset neurotoxicity with oral use of methotrexate is very rare. We report a 10-year-old boy presenting with acute onset limb weakness and neurological signs due to methotrexate-induced leukoencephalopathy following oral methotrexate.

## Case presentation

A 10-year-old Sri Lankan boy presented with fever and headache for 5 days and difficulty in walking for 2 days. The fever was low grade and appeared on and off; however, the child had been well between fever spikes. He did not complain of photophobia or vomiting. When the fever subsided, he developed an imbalance in walking, which gradually worsened over the next 2 days. He was unable to stand unaided on admission. His parents complained of repetitive, involuntary extension movements involving the right upper limb. There was no slurring of speech, difficulty in swallowing, urinary incontinence, or fecal incontinence.

He was previously diagnosed with acute lymphoblastic leukemia at the age of 3 years when he presented with bone pain and difficulty in walking. The leukemia remission was achieved following chemotherapy; however, he developed a central nervous system relapse at 6 years. At admission, he was on treatment for this relapse with daily oral dexamethasone and mercaptopurine and weekly oral methotrexate and folinic acid. He was also on intrathecal vincristine once every two weeks.

On examination, he was afebrile, conscious, and rational. His Glasgow coma scale (GCS) score on admission was 15/15. There was no neck stiffness, and the Kernig sign was negative. Nervous system examination revealed dystonic movements of the right upper limb and hypotonia and reduced muscle power (grade 3/5) of the left upper and lower limbs proximally and distally. The muscle power of the right side was grade 4 (out of 5). Tendon reflexes were diminished in all four limbs, and the plantar response was flexor bilaterally. The child had dysmetria and intension tremors on both sides; however, there were no other cerebellar signs. Examination of pupils, fundi, and cranial nerves were normal. His was hemodynamically stable with a pulse rate of 88 beats/minute and blood pressure of 124/80 mmHg. Respiratory system and abdominal examinations were normal.

**Fig. 1:**
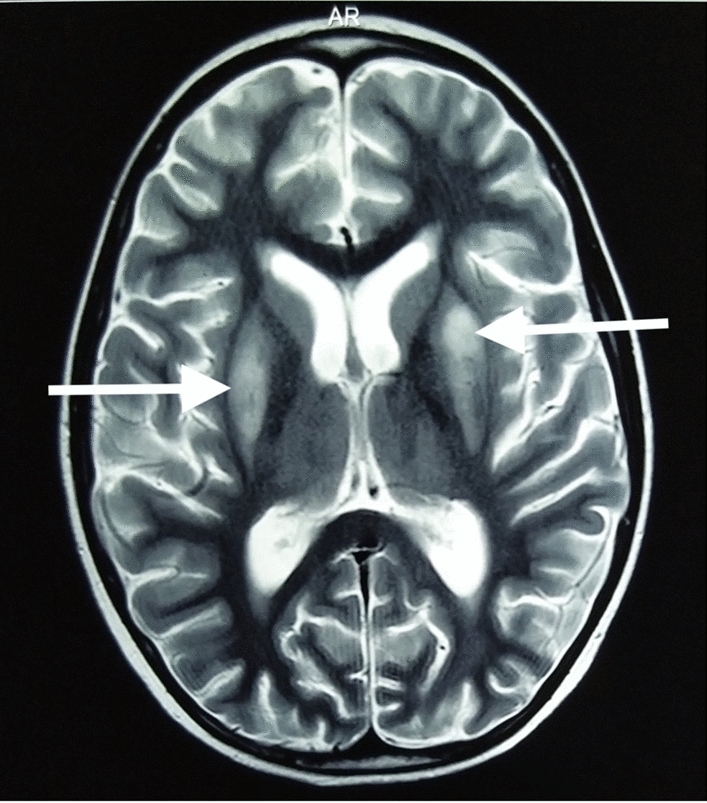
Brain MRI showing almost symmetrical T2 high signal intensities with diffusion restriction of bilateral putamen (arrow) and subcortical areas and deep white matter

Basic hematological and biochemical investigations revealed that his hemoglobin was 11.9 g/dL, white cell count was 4200/µL, platelet count was 156,000/µL, alanine transaminase was 32 IU/L, creatinine was 48 µmol/L, sodium was 140 mmol/L, potassium was 4.5 mmol/L, C-reactive protein was 11 mg/L, and lactate dehydrogenase was 279 IU/L. Non-contrast computed tomography (CT) of the brain did not show evidence of cerebral edema, hemorrhage, or infarction. Due to the diagnostic confusion, urgent magnetic resonance imaging (MRI) of the brain was arranged within 12 hours of admission. T2-weighted MRI revealed symmetrical high signal intensities with diffusion restriction involving bilateral putamen, subcortical areas, and deep white matter (Fig. 1). The MRI appearance was in favor of treatment-related neurotoxicity. On the basis of the clinical features and supportive MRI evidence, treatment-related neurotoxicity due to methotrexate-induced leukoencephalopathy was diagnosed.

The child was started on intravenous folinic acid, aminophylline, dexamethasone, and oral dextromethorphan. Oral methotrexate was discontinued. He was also given intravenous cefotaxime and acyclovir. His clinical conditions worsened during the first 3 days of treatment with autonomic instability (fluctuating heart rate and blood pressure), confusion, and slurred speech. His GCS deteriorated to 9/15. He was briefly admitted to the pediatric intensive care unit for monitoring and supportive treatment, and he then showed gradual improvement in limb weakness and other neurological signs. He was discharged home after 2 weeks.

## Discussion

Methotrexate is an anti-folate medication with anti-neoplastic properties that is used in the treatment of many cancers, including leukemia and lymphoma. It can be administered orally, intravenously, or intrathecally for various indications. One recognized adverse effect of methotrexate is its neurotoxic effects. Neurotoxicity of methotrexate can manifest as aseptic meningitis, transverse myelopathy, encephalopathy, or leukoencephalopathy.

Methotrexate-induced leukoencephalopathy is characterized by white matter hyperintensities on T2-weighted MRI. The recognized clinical features are gradual impairment of cognitive function, learning disability, drowsiness, seizures, ataxia, and hemiparesis [7]. A prospective study of 369 children with acute leukemia treated with methotrexate reported that 14 (3.8%) patients developed clinical neurotoxicity, of which 13 had leukoencephalopathy [8]. The risk of leukoencephalopathy was dose-dependent, and an increased prevalence was observed in patients treated with higher doses of methotrexate than with lower doses [9].

The clinical features of leukoencephalopathy usually follow an insidious onset and slow progression over weeks to months [6]. In contrast, the patient described in this case report showed an unusually rapid course of illness, and the clinical features evolved speedily over 1–2 days. The reason for the rapid onset of our patient’s symptoms is poorly explained. It could either be due to a genetic predisposition of the patient or a yet unexplained mechanism causing neurotoxicity.

Another unusual feature in this case report is the development of leukoencephalopathy following oral methotrexate. Most previous reports of methotrexate-induced leukoencephalopathy followed intrathecal or intravenous use of the drug. Other risk factors for methotrexate-induced leukoencephalopathy are central nervous system radiotherapy and previous treatment with intrathecal or intravenous methotrexate. Our patient did not receive radiotherapy or intrathecal methotrexate, and the leukoencephalopathy was solely due to the oral use of methotrexate [8].

The diagnosis of methotrexate-induced leukoencephalopathy is challenging. As clinical features are non-specific, the definitive diagnosis is dependent on neuroimaging. The characteristic MRI feature is high signal intensity in white matter in T2-weighted images [10]. Our patient had T2 high signal intensities with diffusion restriction of bilateral putamen and subcortical areas and deep white matter areas confirming the diagnosis of methotrexate-induced leukoencephalopathy.

The treatment of methotrexate-induced leukoencephalopathy is still evolving and is guided by case reports [11]. Our patient was treated with folinic acid, aminophylline, dexamethasone, and dextromethorphan, in addition to discontinuation of methotrexate. Exogenous folinic acid is believed to bypass the inhibition of the conversion of folic acid to folinic acid by methotrexate and restores nucleic acid synthesis. Aminophylline acts by reducing the cerebrospinal fluid adenosine concentration that causes vasodilatation in the brain and leads to cerebral ischemia [12]. The rationale for using dextromethorphan is to antagonize the action of elevated homocysteine caused by methotrexate on the *N*-methyl-D-aspartate receptors [13]. Our patient showed improvement within days of starting treatment and was discharged home after 2 weeks.

## Conclusion

In conclusion, this case report describes a patient with rapidly progressing methotrexate-induced leukoencephalopathy following oral methotrexate. It highlights that the risk of neurotoxicity persists even with the oral use of methotrexate; therefore, the prescribers should be vigilant of this uncommon side effect. It also emphasizes the importance of high clinical suspicion and the value of timely neuroimaging to arrive at the proper diagnosis in situations of diagnostic uncertainties.

## Data Availability

Not applicable.

## Abbreviations

MRI: Magnetic resonance imaging
CT: Computed tomography
GCS: Glasgow coma scale
